# Modularity in Motor Control: Similarities in Kinematic Synergies Across Varying Locomotion Tasks

**DOI:** 10.3389/fspor.2020.596063

**Published:** 2020-11-13

**Authors:** Bernd J. Stetter, Michael Herzog, Felix Möhler, Stefan Sell, Thorsten Stein

**Affiliations:** ^1^Institute of Sports and Sports Science, Karlsruhe Institute of Technology, Karlsruhe, Germany; ^2^Joint Center Black Forest, Hospital Neuenbuerg, Neuenbuerg, Germany

**Keywords:** motor coordination, movement organization, principal component analysis, full body kinematics, everyday locomotion tasks

## Abstract

Kinematic synergies (kSYN) provide an approach to quantify the covariation of joint motions and to explain the mechanisms underlying human motor behavior. A low-dimensional control strategy by means of the activation of a moderate number of kSYN would simplify the performance of complex motor tasks. The purpose of this study was to examine similarities between the kSYN of varying locomotion tasks: straight-line walking, walking a 90° spin turn and walking upstairs. Task-specific kSYN were extracted from full body kinematic recordings of 13 participants by principal component analysis. The first five kSYN accounting for most of the variance within each task were selected for further analysis following previous studies. The similarities between the kSYN of the three different locomotion tasks were quantified by calculating cosine similarities (SIM), as a vector-based similarity measure ranging from 0 (no similarity) to 1 (high similarity), between absolute principal component loading vectors. A SIM between two kSYN > 0.8 was interpreted as highly similar. Two to three highly similar kSYN were identified when comparing two individual tasks with each other. One kSYN, primarily characterized by anteversion and retroversion of the arms and legs, were found to be similar in all three tasks. Additional kSYN that occurred between individual tasks reflected mainly an upwards/downwards movement of the body or a countercyclical knee flexion/extension. The results demonstrate that the three investigated locomotion tasks are characterized by kSYN and that certain kSYN repeatedly occur across the three locomotion tasks. PCA yields kSYN which are in descent order according to their amount of total variance accounted for. Referring to the placing of a kSYN within the order as priorization, we found a change in priorization of repeatedly occurring kSYN across the individual tasks. The findings support the idea that movements can be efficiently performed through a flexible combination of a lower number of control-relevant variables.

## Introduction

The true complexity of the control processes involved in ordinary human movements is masked by the ease of their execution (Wolpert et al., 2013). The human central nervous system (CNS) consists of billions of interconnected neurons, and the musculoskeletal system is composed of approximately 700 muscles and over 300 mechanical degrees of freedom (Bruton and O'Dwyer, 2018). This highly redundant motor system enables us to achieve movement in countless ways (Bernstein, 1967), and one of the longstanding questions in motor control research is how the CNS resolves this redundancy. In addition, we learn an enormous number of skills, such as raising a hand or playing sports, in the course of our lives and even when the execution of such tasks seems to be easy, it requires a fine tuning of the CNS. This leads to another fundamental question of motor control research: namely, how this versatility is implemented in the CNS. Consequently, finding answers to these two questions through analyzing the coordination of human movements, has—besides other challenges such as dealing with non-linearities within the motor system (Franklin and Wolpert, 2011)—become a central issue in motor control research (e.g., Bizzi et al., 1991; Wolpert and Kawato, 1998; Scholz and Schöner, 1999; Todorov and Jordan, 2002; d'Avella et al., 2003; Daffertshofer et al., 2004; Lacquaniti et al., 2012).

A possible answer to the questions of how the CNS solves the challenge of versatility and redundancy could be through a modular control architecture (Wolpert and Kawato, 1998; d'Avella, 2016). Many daily tasks are not independent from each other and have certain similarities, e.g., walking straight ahead vs. walking in a curve. If motor skills are represented by a collection of compositional elements (Giszter, 2015; d'Avella, 2016) that act as building blocks for movement construction, one would assume that similar movement tasks (e.g., walking in a straight line vs. walking in a curve) are composed of similar elements, although these may be weighted differently during construction of movements.

Such compositional elements could have various forms (Giszter, 2015). Synergies have been proposed as one possibility for implementing the idea of a modular control architecture (Bizzi et al., 1991; Bruton and O'Dwyer, 2018). Synergies ensure organization by establishing working relationships and thus simplifying the control of movements in a highly redundant motor system (Bernstein, 1967; Bruton and O'Dwyer, 2018). Such synergies can either exist on a muscular (d'Avella et al., 2003) or kinematic level (Borghese et al., 1996; Catavitello et al., 2018) and they typically represent compositional elements working together to produce results not obtainable by any of the elements alone (McGowan et al., 2010; Wang et al., 2013; Tagliabue et al., 2015). There is a growing body of literature supporting the existence of synergies and demonstrating that multi-segmental movements are highly coupled and correlated for a variety of tasks (Kelso et al., 1983; Lacquaniti et al., 1986; Troje, 2002; Daffertshofer et al., 2004; Wang et al., 2013; Majed et al., 2017; Haid et al., 2018).

The use of principal component analysis (PCA) has been proven to be effective in reducing the redundancy of large kinematic datasets and has been shown to be a feasible approach to extract relevant hidden structures (Courtine et al., 2005; Wang et al., 2013; Majed et al., 2017; Zago et al., 2017b; Bruton and O'Dwyer, 2018). Such analysis performed on a full body kinematic dataset decomposes the complex movement pattern into its main kinematic synergies (kSYN) (Daffertshofer et al., 2004; Lamoth et al., 2009; Wang et al., 2013). The first several few principal components normally account for most of the variance in the original data, and can be interpreted as the kinematic elements by which the motor system organizes a movement (Wang et al., 2013). For example, most previous studies stated that the dimensionality of gait could be reduced to 3–5 kSYN (Courtine and Schieppati, 2004; Wang et al., 2013; Zago et al., 2017c). Within the last two decades, kSYN of whole body motion have been investigated for common locomotion tasks such as walking or running (Troje, 2002; Daffertshofer et al., 2004; Lamoth et al., 2009; Federolf et al., 2013); balance tasks (Federolf, 2016; Haid et al., 2018); and more complex movements such as contemporary dance (Hollands et al., 2004) or karate (Zago et al., 2017a). In the majority of the studies, redundancies and patterns of coordination were determined in order to gain insight into the movement control mechanisms (Wang et al., 2013).

One of the first studies investigating basic coordination patterns in straight-line walking and walking turns using PCA was done by Courtine and Schieppati (2004). Their findings indicated invariant coordination patterns among limb segments and the trunk during straight-line walking and walking turns. Furthermore, a turn-dependent tuning of the coordination patterns was observed depending on the walking direction of the body. Such adaptations are required to successfully turn, as the center of mass must be quickly halted and redirected over a relatively stable base of support (Dixon et al., 2013). The investigation of turning biomechanics showed changes in lower-limb joint kinematics and spatio-temporal differences for the two main turning strategies for 90° turns, namely spin turn and step turns (Taylor et al., 2005; Dixon et al., 2013). The spin turn is characterized by a change of direction toward the same side as the stance limb and has been postulated to be an economical turning strategy (Taylor et al., 2005; Dixon et al., 2013). Similarly, other frequently encountered locomotion tasks by humans in daily living, such as stair walking, are characterized by biomechanical changes in comparison to straight-line walking. Riener et al. (2002) described a greater knee angle and the change from heel contact to middle food contact while walking stairs and hypothesized that the participants switch their gait patterns. Whether such changes lead to a similar tuning of kSYN as proposed for walking turns has not been examined so far. Overall, differences in coordination due to changes in the locomotion task have not been studied extensively. A few studies analyzed similarities in whole body kSYN. Lamoth et al. (2009) examined whole body kinematics when comparing multi-segmental coordination and stride characteristics in walking and running. They highlighted that “walking and running entail similar, albeit speed- and gait-dependent, coordination structures.” Their finding suggested that similar neural circuits in the spinal cord control the two locomotion tasks walking and running (Lamoth et al., 2009). d'Avella et al. (2003) came to a similar conclusion when they related muscle activity patterns to movement kinematics in frogs. They proposed the “existence of a substantial amount of shared structure in the control of different tasks” as well as “the existence of behavior-specific synergies.” They concluded that mixing behavior-independent and behavior-specific modules allows for the execution of different, complex behaviors. Overall, the link between muscle synergies, kSYN and movement production can be described as follow: a certain set of muscle synergies are required to produce a movement and the consequence of activating muscle synergies leads to the activation of the associated kSYN. This leads to the assumption that the CNS can benefit from a flexible combination of kSYN as supplements to muscular synergies for a range of similar movements.

Taken together, how and to what extend whole body kSYN are utilized across varying locomotion tasks has not yet been studied extensively. However, a deeper understanding on a cross-task use of whole body kSYN can help to better understand how the CNS takes advantage of a modular control architecture to efficiently solve the degrees of freedom problem in locomotion tasks. Therefore, the purpose of this study was to examine similarities in whole body kSYN between varying locomotion tasks by investigating straight-line walking, walking a 90° spin turn and walking upstairs. We expected that: (1) characteristic kSYN for the three tasks are identifiable and (2) certain kSYN repeatedly occur across the three tasks due to their similarity.

## Materials and Methods

### Participants

Thirteen male volunteers (age 26.1 ± 2.9 years; height 178.7 ± 5.5 cm; body mass 78.4 ± 5.9 kg) participated in this study. All participants were physically active and had no known history of neurological or motor disorders or injuries over the last 6 months. The study was approved by the ethics committee of the Karlsruhe Institute of Technology. All participants were informed of the experimental procedures and gave informed written consent prior to study participation.

### Data Acquisition

Full body kinematic data of straight-line walking (SW), walking a 90° spin turn (WT), and walking upstairs (WU), as tasks among the most common forms of human gait (Riener et al., 2002; Glaister et al., 2007), were collected using a marker-based motion capture system (Vicon Motion Systems Ltd., Oxford, UK) with a sampling rate of 200 Hz. Eighteen passive-reflective markers were placed bilaterally on the participants' forehead, shoulder, elbow, hand, pelvis, knee, ankle, heel, and forefoot following previous studies (Daffertshofer et al., 2004; Federolf et al., 2013). SW trials were collected while participants walked overground. For the WT trial, participants followed a path with a 90° curve to the right drawn on the ground. They were instructed to use a spin turn strategy, which means that they had to perform their first turning step with the left foot (Taylor et al., 2005). A staircase of seven steps was used to collect the WU trials. The stair tread had a height of 0.17 m, which is right in the middle of the DIN-normed range for stair treads (DIN 18065). All locomotion tasks were performed at a self-selected speed. Prior to the recording of one valid trial for each specific locomotion task, participants were given two to three practice trials per locomotion task.

### Data Processing

Vicon Nexus software (V. 1.8.5, Oxford, UK) was used to produce gap-free 3D marker trajectories. Further data processing steps were carried out in Matlab (The MathWorks Inc., Natick, MA, USA). 3D marker trajectories were low-pass filtered (Butterworth 4th order) at a cut-off frequency of 15 Hz. Gait cycles for the left leg were identified by determining initial contact as the minimum of the vertical heel marker trajectories for SW and WT. The minimum of the forefoot marker was determined to identify gait cycles during WU, as the initial contact with the stair was made with the forefoot (Riener et al., 2002). Data were extracted from the gait cycle at the mid-point of the walkway, the turning step, in the middle of the three turning phases of approach, turning and departure (Dixon et al., 2013), and at the mid-point of the stairs as representative for SW, WT, and WU, respectively. Gait cycles were time-normalized to 100 data points. All marker coordinates were expressed relative to the horizontal position of the pelvis, i.e., the horizontal center of the pelvis markers was subtracted from all marker coordinates. To minimize the influence of anthropometric differences on the calculation of the kSYN, the mean over the analyzed period was subtracted and the marker trajectories were normalized to unit standard deviation (Daffertshofer et al., 2004). Based on the normalized marker trajectories, a movement data matrix was formed for each task. The dimension of the matrices was 1,300 (13 participants × 100 time points) × 54 (18 markers × 3D coordinates).

### Extraction of Kinematic Synergies

**Figure 1:**
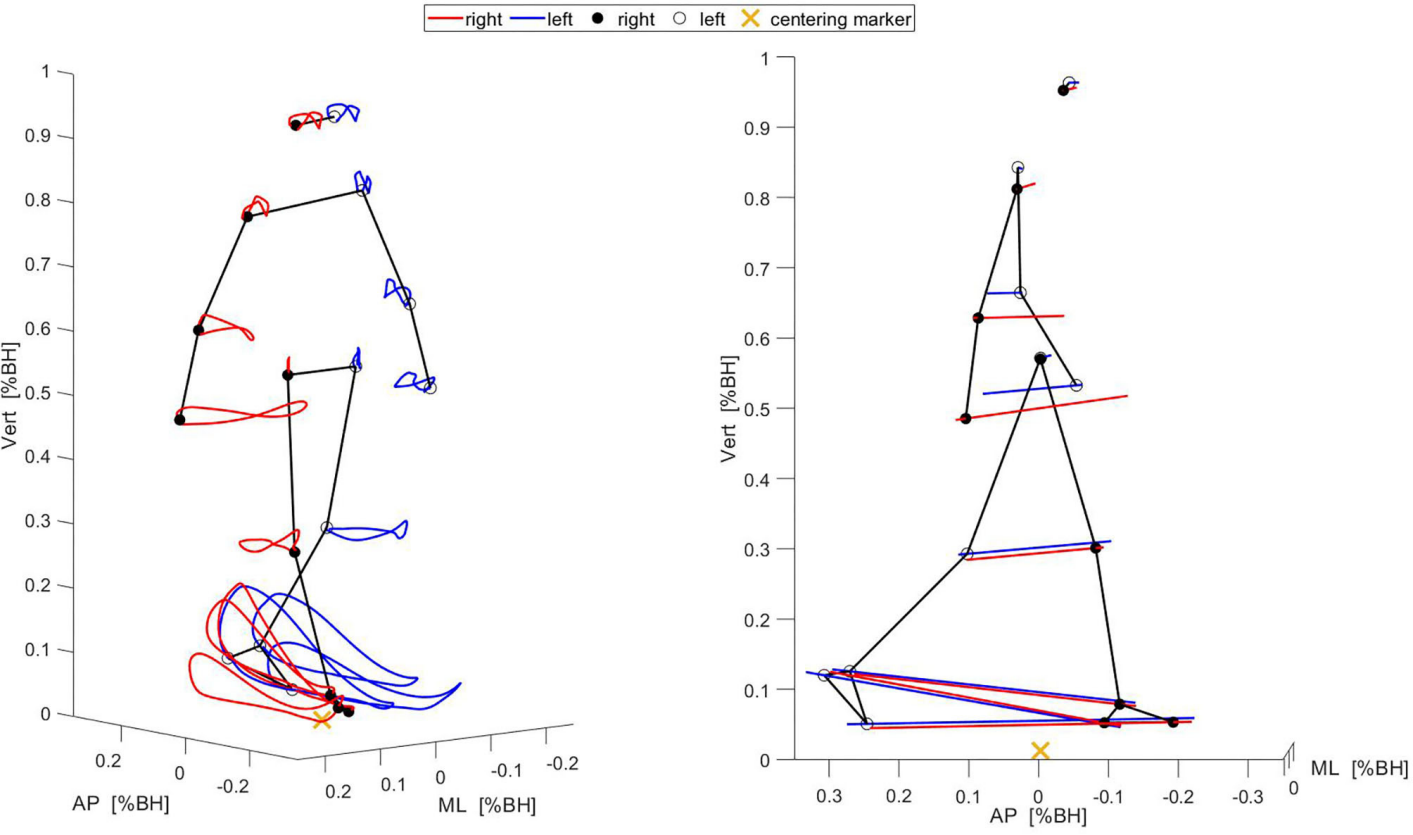
Illustration of the full movement (Left) and the first kSYN (Right) of one gait cycle while the participant walked in a straight line. The stick figures show the marker positions at the beginning of the gait cycle. The red and blue lines show the marker trajectories over the whole gait cycle. AP, anterior-posterior; ML, medial-lateral; Vert, vertical; BH, body height.

kSYN for each task were extracted by applying PCA to the corresponding movement data matrices. PCA was performed using singular value decomposition. Each PCA yielded (i) principal component vectors *PC*_*k*_, (ii) eigenvalues *EV*_*k*_, and (iii) scores (Daffertshofer et al., 2004; Federolf et al., 2013; Wang et al., 2013). The *PC*_*k*_ indicate the directions of the largest variations in the movement data matrix. The eigenvalues indicate the fraction of the total variance accounted for by each *PC*_*k*_. The scores contain the projections of the original movement data onto each *PC*_*k*_. *k* denotes the order of the eigenvectors. The components, i.e., loadings of the *PC*_*k*_, quantify the contribution of the original variables (1D marker coordinates) to a specific kSYN (Esbensen et al., 2002). A high loading value indicates that this variable strongly loads on a particular kSYN. Loadings were expressed as absolute values. To provide an intuitive interpretation of the kSYN, they were visualized as two-dimensional stick figures in the original marker coordinates (Federolf et al., 2012; Haid et al., 2018). This included the projection of individual scores on specific *PC*_*k*_ and the rescinding of the normalization (multiply by the standard deviation of the time series and add mean of the time series). Consequently, isolated deviations from the mean body position of an individual caused by a single kSYN could be represented graphically. Figure 1 exemplifies the full movement as well as the first extracted kSYN of SW for one participant.

### Similarity Analysis

The similarities between the first five kSYN according to their eigenvalues (Wang et al., 2013; Zago et al., 2017c) of the three different locomotion tasks were quantified using cosine similarity (SIM; Singh et al., 2018). SIM is a vector-based similarity measure ranging from 0 (no similarity) to 1 (high similarity), as long as all components of the vector are positive (Zhang, 2008). A SIM between two kSYN > 0.8 is interpreted as highly similar (Xiao et al., 2008; Song and Chen, 2013; Saito et al., 2018). The SIM between two kSYN is calculated as follows:

$$\begin{array}{l} SIM\left(\vec{PC_{n}},\vec{PC_{m}}\right)=\frac{\sum_{i=1}^{54}PC_{n,i}\;*\;PC_{m,i}}{\sqrt{\sum_{i=1}^{54}{PC_{n,i}}^{2}}\;*\;\sqrt{\sum_{i=1}^{54}{PC_{m,i}}^{2}}} \end{array}$$

where $\vec{PC_{n}}$ and $\vec{PC_{m}}$ refer to the corresponding principal component loading vectors of the two kSYN under comparison, and *i* indicates the vector component. According to the equation, two kSYN were considered similar if the same variables load equally on both kSYN, and this is reflected in a high SIM value. SIM has been shown to produce high quality results across different fields (Lee et al., 2011; Xhafa et al., 2014).

## Results

### Identified Kinematic Synergies

The aspects of the whole movement represented by each kSYN are listed in Table 1 and visualized in the videos submitted as Supplementary Materials. Together, the first five kSYN explained 83.9, 91.1, and 86.5% of the variance in the kinematic data for SW, WT, and WU, respectively.

**Table 1:** Description of the first five kinematic synergies (kSYN) of the three different locomotion tasks.

**kSYN**	**EV [%]**	**Characterization**	**SIM > 0.80**
**STRAIGHT-LINE WALKING (SW)**			
SW1	37.0	Anteversion and retroversion of the arms and legs	WU2 (0.85), WT2 (0.84)
SW2	21.6	Countercyclical knee flexion/extension with countercyclical rising and lowering of the heels	WU3 (0.84)
SW3	15.9	Upwards/downwards movement of the body	WT3 (0.89)
SW4	5.4	Cyclical knee flexion/extension with anteversion/retroversion of the arm	WU5 (0.83)
SW5	4.0	Cyclical knee flexion/extension with hip flexion/extension	
**WALKING 90°** **SPIN TURN (WT)**			
WT1	43.1	Whole body rotation around the longitudinal axis	
WT2	23.3	Anteversion and retroversion of the arms and legs	SW1 (0.84), WU2 (0.86)
WT3	14.4	Upwards/downwards movement of the body with unilateral knee flexion	SW3 (0.89), WU1 (0.84)
WT4	8.1	Knee flexion of the swing leg with minor upper body rotation around the longitudinal axis	
WT5	2.2	Whole body rotation around the longitudinal axis with synchronous knee flexion/extension	
**WALKING UPSTAIRS (WU)**			
WU1	39.8	Upwards movement of the body with unilateral knee flexion	WT3 (0.84)
WU2	28.9	Anteversion and retroversion of the arms and legs	WT2 (0.86), SW1 (0.85)
WU3	9.6	Countercyclical knee flexion/extension with upwards movement of the body	SW2 (0.84)
WU4	4.5	Forward/backward leaning of the upper body	
WU5	3.7	Synchronous knee and arm flexion	SW4 (0.83)

*The eigenvalues (EV) indicate the fraction of the total variance accounted for by each kSYN. The right-hand column shows the highly similar kSYN (cosine similarity (SIM) >0.80) across the different locomotion tasks*.

### Similarities Between Kinematic Synergies

SW and WT showed two highly similar kSYN (Table 1): one between SW3 and WT3 (SIM = 0.89) and the second between SW1 and WT2 (SIM = 0.84). All other comparisons between SW and WT yielded SIM <0.79.

SW and WU showed three highly similar kSYN (Table 1): one between SW1 and WU2 (SIM = 0.85), the second between SW2 and WU3 (SIM = 0.84), and the third between SW4 and WU5 (SIM = 0.83). All other comparisons between SW and WU yielded SIM <0.77.

WU and WT showed two highly similar kSYN (Table 1): one between WU2 and WT2 (SIM = 0.86) and the second between WU1 and WT3 (SIM = 0.84). All other comparisons between WT and WU yielded SIM <0.78.

**Figure 2:**
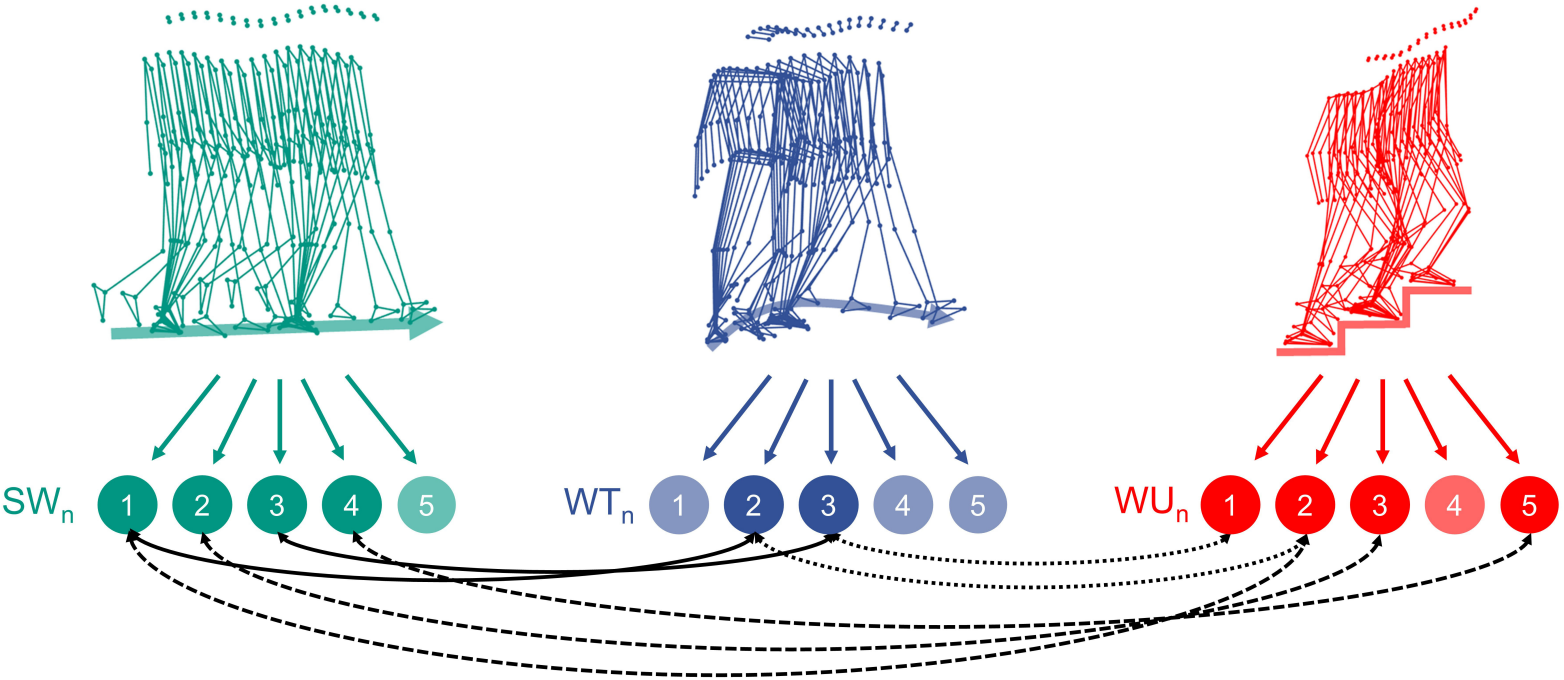
Schematic representation of the three locomotion tasks (SW, straight-line walking; WT, walking 90° spin turn; WU, walking upstairs) and their decomposition into five main kinematic synergies. The black arrows highlight high similarities (cosine similarity >0.80) across the different locomotion tasks.

Figure 2 illustrates similarities between the kinematic synergies of the three investigated locomotion tasks.

## Discussion

The current study examined similarities in kSYN across three common locomotion tasks: straight-line walking, walking a 90° spin turn and walking upstairs. Previous research in motor control has highlighted that the CNS organizes movement by flexible combinations of a low number of synergies (d'Avella, 2016; Lambert-Shirzad and van der Loos, 2017; Bruton and O'Dwyer, 2018). Based on the current literature, we expected that: (1) we could identify characteristic kSYN for the three tasks and (2) certain kSYN would repeatedly occur across the three tasks due to the similarity of the locomotion involved. For this purpose, similarities in kSYN were compared across the three tasks. The study revealed that (1) the first five kSYN accounted for more than 83.9% of the total variance of each task and (2) two to three kSYN were in common across the three tasks, while other kSYN were task specific. Common kSYN across the three tasks predominantly represented the anteversion and retroversion of the arms and legs, the upwards/downwards movement of the body and flexion/extension movements of the knees. In consequence, the results confirm our hypotheses and help to gain a deeper understanding on the construction of locomotion movements.

### Identification of Kinematic Synergies

Previous studies investigating whole body kinematics showed a reduction of control-relevant degrees of freedom via kSYN for various tasks including locomotion (Troje, 2002; Daffertshofer et al., 2004; Lamoth et al., 2009; Federolf et al., 2013), balance tasks (Federolf, 2016; Haid et al., 2018), and more complex movements (Hollands et al., 2004; Zago et al., 2017a). In line with related studies on whole body gait patterns for straight-line walking (Daffertshofer et al., 2004; Majed et al., 2017), we found a small number of compositional elements that described the essential features of gait: a combination of five kSYN explained 83.9% of the variance in the kinematic data. A comparable reduction to control relevant degrees of freedom during curved walking was reported by Courtine and Schieppati (2004) when they analyzed the limb segments and the trunk. Similarly, in an earlier study, Borghese et al. (1996) described the existence of laws of intersegmental coordination when investigating lower limb kinematics during walking. The authors observed regular loops on a plane for the elevation angles of the limb segment (pelvis, thigh, shank, and foot), despite large excursions of the individual angles, across six males. Lacquaniti et al. (2012) further reported in their review on patterned control of human locomotion that the so-called planar covariance corresponds with muscle activation patterns in order to simplify the problem of control of multi-segmental movements. Studies at the muscular level also suggest that movement tasks are performed using combined synergies (Cappellini et al., 2006; Bejarano et al., 2017; Bruton and O'Dwyer, 2018; Maguire et al., 2019). Additionally, animal studies support that the nervous system may use global variables having fewer degrees of freedom for controlling locomotion (Ivanenko et al., 2007; Catavitello et al., 2018). Catavitello et al. (2018) underlined the existence of kSYN when investigating the planar covariation of limb segment motion across a wide range of animals in a more recent study. The authors stated that kSYN lie at the interface between neural command signals and the mechanics of locomotion. A closer look at the first five kSYN of the three analyzed tasks (Table 1) highlights the phenomenon described by Daffertshofer et al. (2004) that these components reflect movements oscillating at either the stride frequency (e.g., arm and leg swing) or the second harmonic (i.e., movement components that oscillate at double frequency of the stride frequency, such as knee bending). Our finding supports the idea that the CNS may use kSYN as a possible implementation of the idea of a modular control architecture to deal with the large number of degrees of freedom of the motor system. Moreover, results are in line with the idea of mixing of behavior-independent and behavior-specific modules for the execution of different, complex behaviors, described by d'Avella et al. (2003).

### Occurrence of Kinematic Synergies Across Locomotion Tasks

Regarding a cross-task use of kSYN, our findings show a change in the prioritization of similar kSYN. SW1, as the highest prioritized kSYN of SW, showed high similarity to WT2 and WU2, which is downstream of a presumably more task-specific kSYN, such as the body's rotation around the longitudinal axis for WT and the vertical displacement of the center of mass for WU. Interestingly, kSYN with high similarity showed slight variations in their characteristic depending on the individual task. For example, the kSYN characterizing an upwards/downwards movement of the body with unilateral knee flexion in WT (WT3) was highly similar with the kSYN characterizing an upwards/downwards movement of the body in SW (SW3) and the kSYN characterizing an upwards movement of the body with unilateral knee flexion in WU (WU1). This finding indicates that the CNS has the capability to tune kSYN with the actual task requirements for a successful realization of the individual movement task.

The results of Lamoth et al. (2009) also indicate a cross-task use of kSYN. The authors concluded that walking and running entail similar kSYN (referred to in their study as coordinative structures), although these kSYN are speed- and gait-dependent. This finding is supported by similar phenomena at the muscular level during walking (Bejarano et al., 2017). Our findings suggest that, for movement construction in locomotion tasks, the CNS optimizes its kSYN selection: slightly adapt certain cross-task kSYN, reflected by a high SIM between tasks, and complement these by adding more task-specific kSYN to successfully realize the whole body movement.

The identified task-specific kSYN can be seen as a means to perform biomechanical subtasks (Maguire et al., 2019). Biomechanical subtasks of locomotion are, for example, generating body support and forward propulsion (McGowan et al., 2010). Previous research provides evidence that individual muscle synergies are associated with specific subtasks (McGowan et al., 2010; Maguire et al., 2019). This association between muscle synergies and specific subtasks is potentially reflected by the corresponding kSYN, which can be seen as representative for the biomechanical subtasks. For example, the kSYN characterizing the upwards/downwards movement of the body (SW3, WT3, and WU1) can be linked to the biomechanical subtask of generating body support. The result of combined activation of such functional units or referent coordinates form the whole movement for a specific motor task (Latash, 2020). Research on the origin of synergies and the linkage between kSYN and muscle synergies have produced results compatible with this idea (Tagliabue et al., 2015; Leo et al., 2016; Latash, 2020). Overall, the finding of this study—that pronounced kSYN repeatedly occur across the three investigated locomotion tasks—supports the idea that the CNS ensures movement organization by flexible combinations of a low number of synergies representing the idea of a modular control architecture.

The lower correlation between the kSYN in SW and WT (two kSYN with similar characteristics) compared to the relationships between the kSYN in SW and WU (three kSYN with similar characteristics) is possibly due to a greater asymmetry in the motion execution in WT. Asymmetries in WT are caused by rotation about the longitudinal axis and are characterized by different stride lengths (Orendurff et al., 2006). Comparable asymmetries are typically not present in WU (Andriacchi et al., 1980). Overall, it must be noted that the three investigated locomotion tasks can be performed with different strategies, e.g., walking a turn as a spin or step turn (Dixon et al., 2013) or walking upstairs using the step-over-step or step-by-step strategy (Reid et al., 2007), and at different velocities. Furthermore, turning is subdivided in three phases, namely approach, turning, and departure, that are characterized by biomechanical differences (Dixon et al., 2013). However, it remains unsolved how such differences in movement execution affect the kSYN structure and further research would be indispensable to obtain a more detailed understanding how the CNS can benefit from a flexible combination of kSYN.

### Limitations

One consideration worth noting is that the kSYN were calculated based on single gait cycles per participant. Whether the incorporation of multiple trials per participant results in slightly different kSYN remains speculative and should be addressed in the future. The rather small and homogeneous group of participants consisting of 13 males potentially limits the outcome with respect to a general explanation for the mechanisms underlying human motor coordination. Nonetheless, future studies may benefit from the presented approach in this study. Whole body movements were reduced to five kSYN, which covered about 85–90% of the variance in the data. Thus, it can be assumed that the major part of relevant movement aspects is considered. Nonetheless, if the remaining 10–15% of the variance also contains relevant kSYN that could simplify the construction of various locomotion tasks cannot be answered. The investigation of higher order kSYN can be difficult, as such components rather represent movement components of the individual movement execution than common movement components across participants and they are more likely to be susceptible to noise (Federolf et al., 2012, 2014; Wang et al., 2013). Another limitation is that the qualitative descriptions of the kSYN represent subjective interpretations. As the interpretation of the kSYN depends on the decomposition method, this is a potential limitation of the study. The applied PCA approach is a linear decomposition method and the use of different matrix factorization methods could result in a different outcome (Lambert-Shirzad and van der Loos, 2017). In addition, when applying the PCA to marker coordinates, the complex, high-dimensional movements of all markers are transformed into a set of one-dimensional movement components (kSYN). These components of movements cannot necessarily be performed in an isolated form by humans, and instead result from a combination of actual movements (Federolf, 2016). Similarly, it must be mentioned that studies using matrix factorization methods to extract synergies reflect biomechanical constraints of the task and their link to underlying neural strategies of motor control has not been fully explored yet (Lambert-Shirzad and van der Loos, 2017). Finally, the extraction of kSYN using matrix factorization algorithms provide a descriptive model of covariations among segment movements.

### Conclusion

In this study we reported on similarities between kSYN of varying tasks. This study demonstrated that SW, WT, and WU are characterized by kSYN and that certain kSYN repeatedly occur across the three locomotion tasks. In addition to a more detailed analysis of the relationship between kinematic and muscular synergies, future interventions should examine whether such synergies serve as a causal explanatory model. This means that, against the background of a modular control architecture, the ease of learning a task depends on its compatibility with existing synergies. The idea of a modular control architecture addresses an important issue on the interface of theory and practice. In the future, a clear understanding of the kSYN structure of everyday and sport movements could affect the design of neurorehabilitation programs or practice protocols in sport trough targeted exercises for regaining and improving motor function.

## Data Availability Statement

The raw data supporting the conclusions of this article will be made available by the authors, without undue reservation.

## Ethics Statement

The studies involving human participants were reviewed and approved by ethics committee of the Karlsruhe Institute of Technology. The patients/participants provided their written informed consent to participate in this study.

## Author Contributions

BS, MH, SS, and TS were involved in the design of the study. BS and MH carried out all data collection and analysis. BS, MH, FM, and TS were involved in the interpretation and discussion of the results. BS took the lead in writing the manuscript. All authors provided critical feedback and contributed to the final manuscript.

## Conflict of Interest

The authors declare that the research was conducted in the absence of any commercial or financial relationships that could be construed as a potential conflict of interest.
