# Supplementary figures and images for: Modularity in Motor Control: Similarities in Kinematic Synergies Across Varying Locomotion Tasks
Source: Front Sports Act Living. 2020 Nov 13;2:596063. doi: 10.3389/fspor.2020.596063 (PMC7739575; doi:10.3389/fspor.2020.596063)

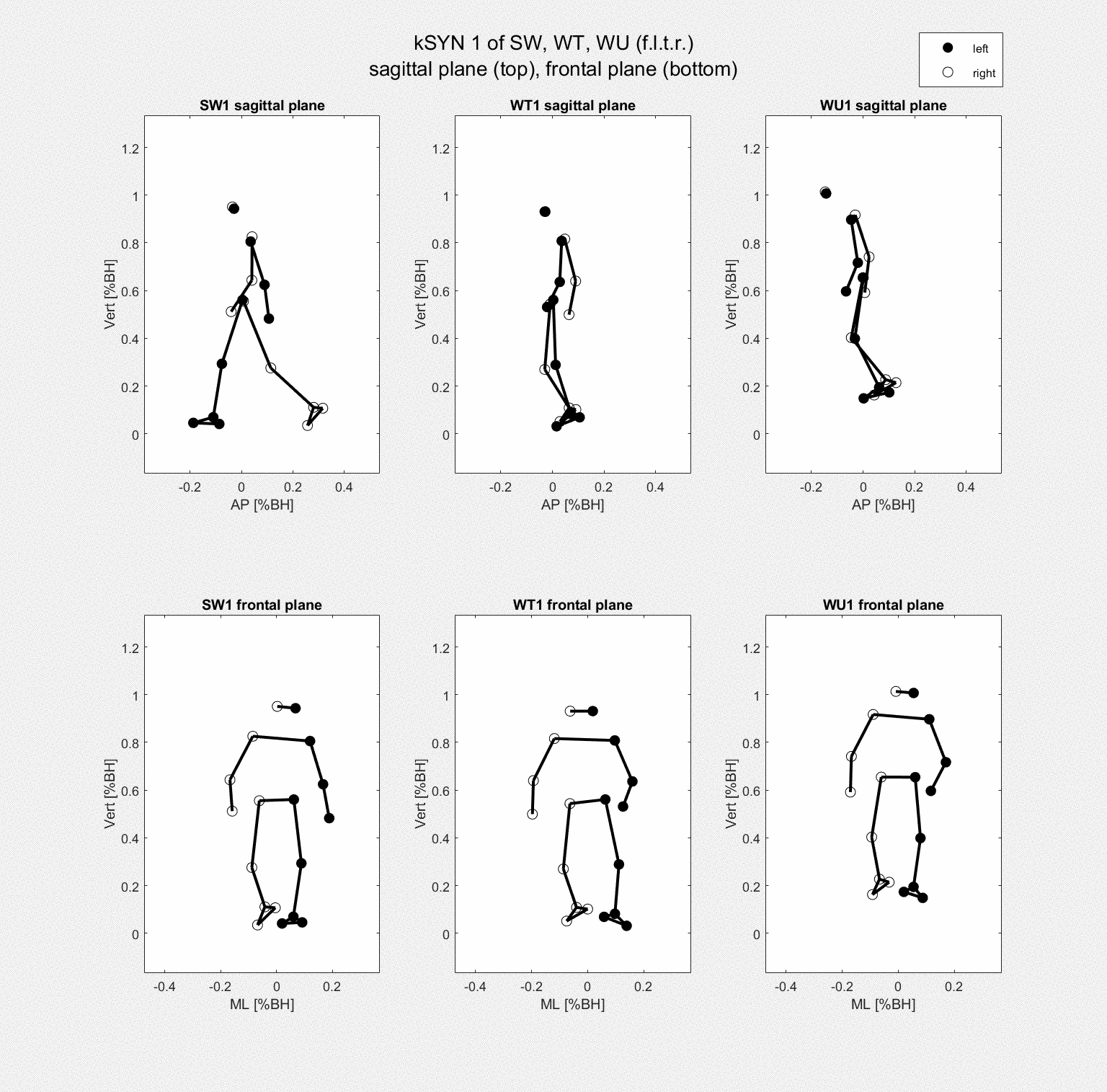

Supplement: Supplementary file 1 [file Data_Sheet_1.zip › kSYN1.gif]

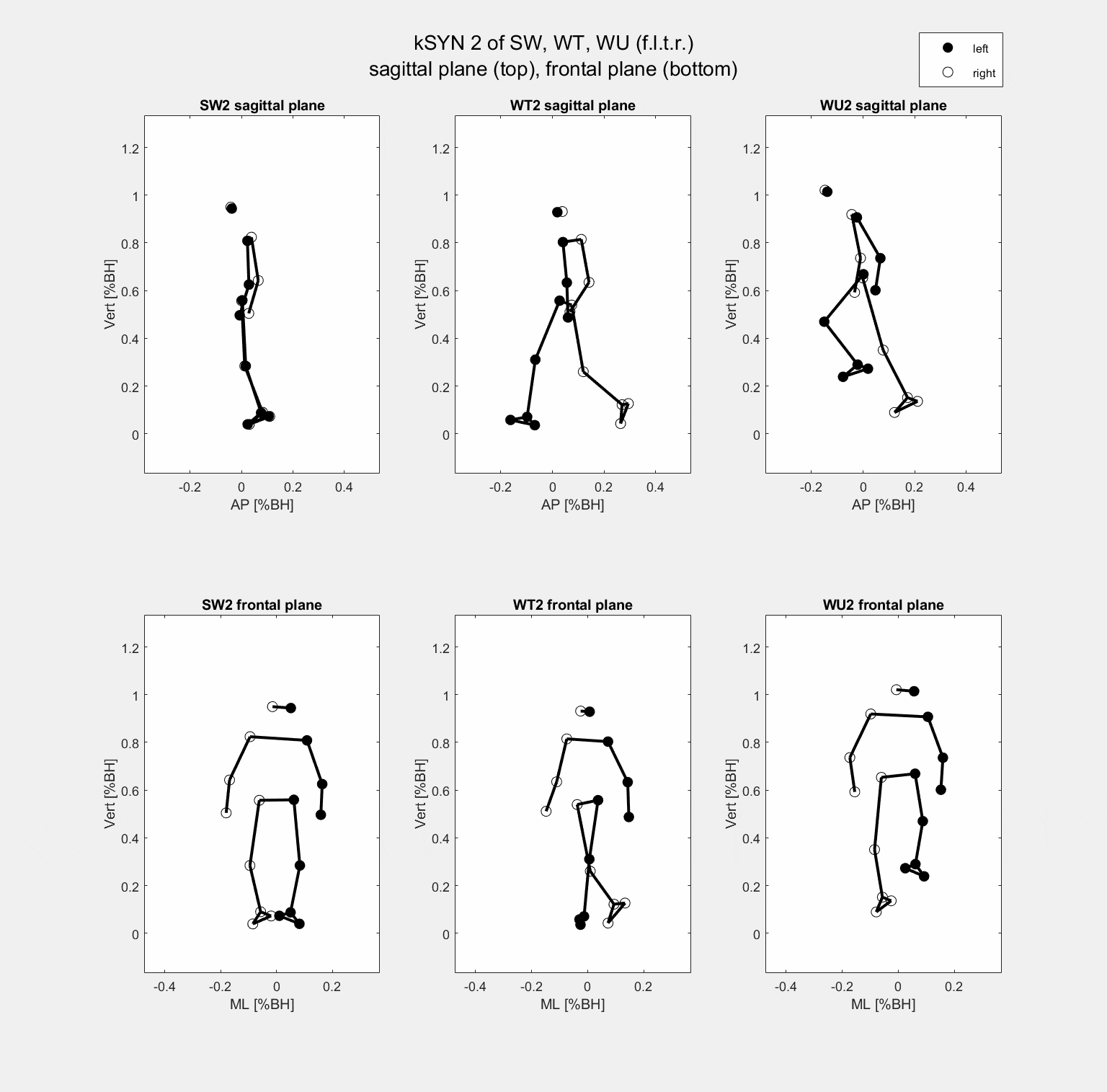

Supplement: Supplementary file 1 [file Data_Sheet_1.zip › kSYN2.gif]

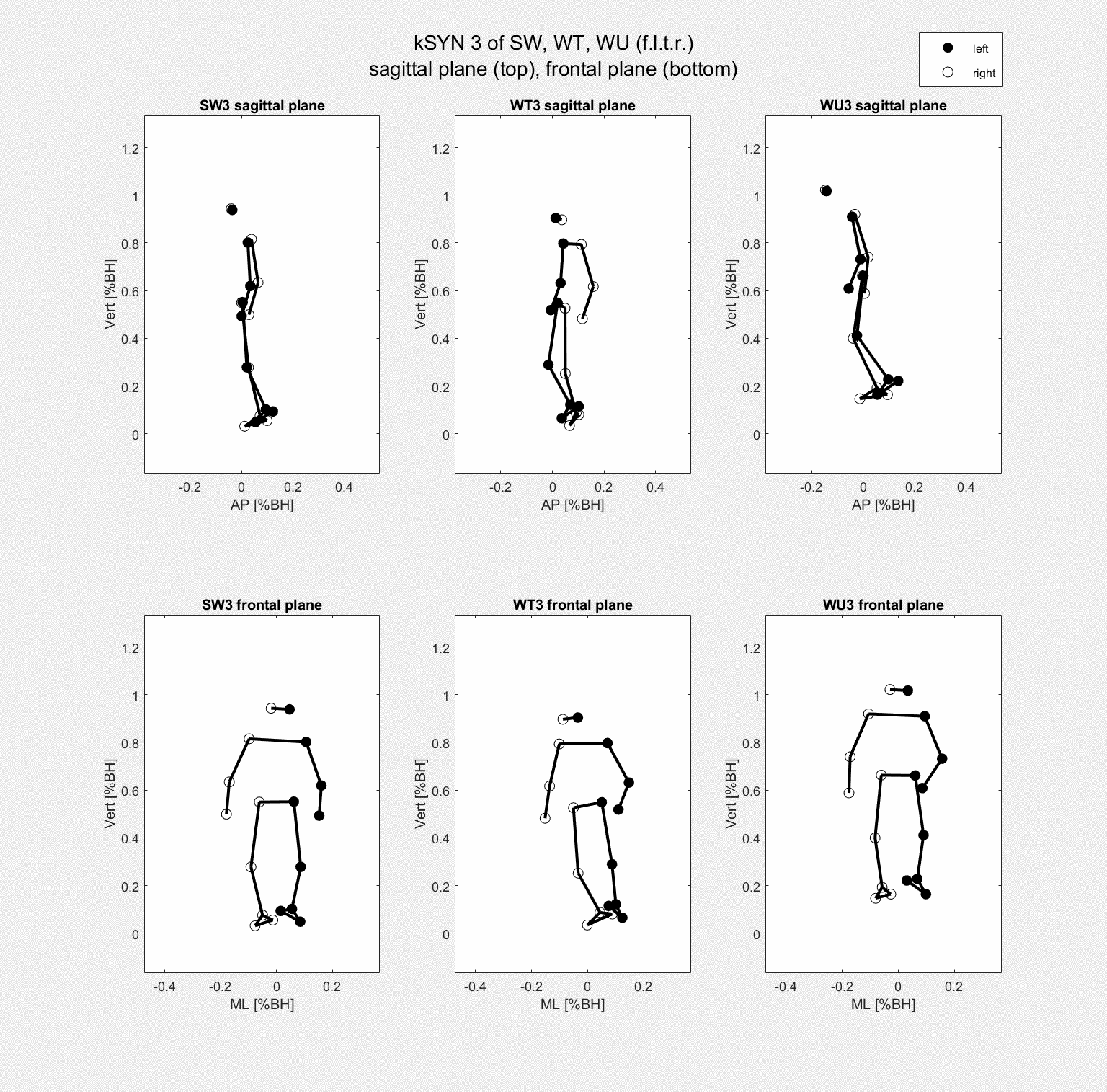

Supplement: Supplementary file 1 [file Data_Sheet_1.zip › kSYN3.gif]

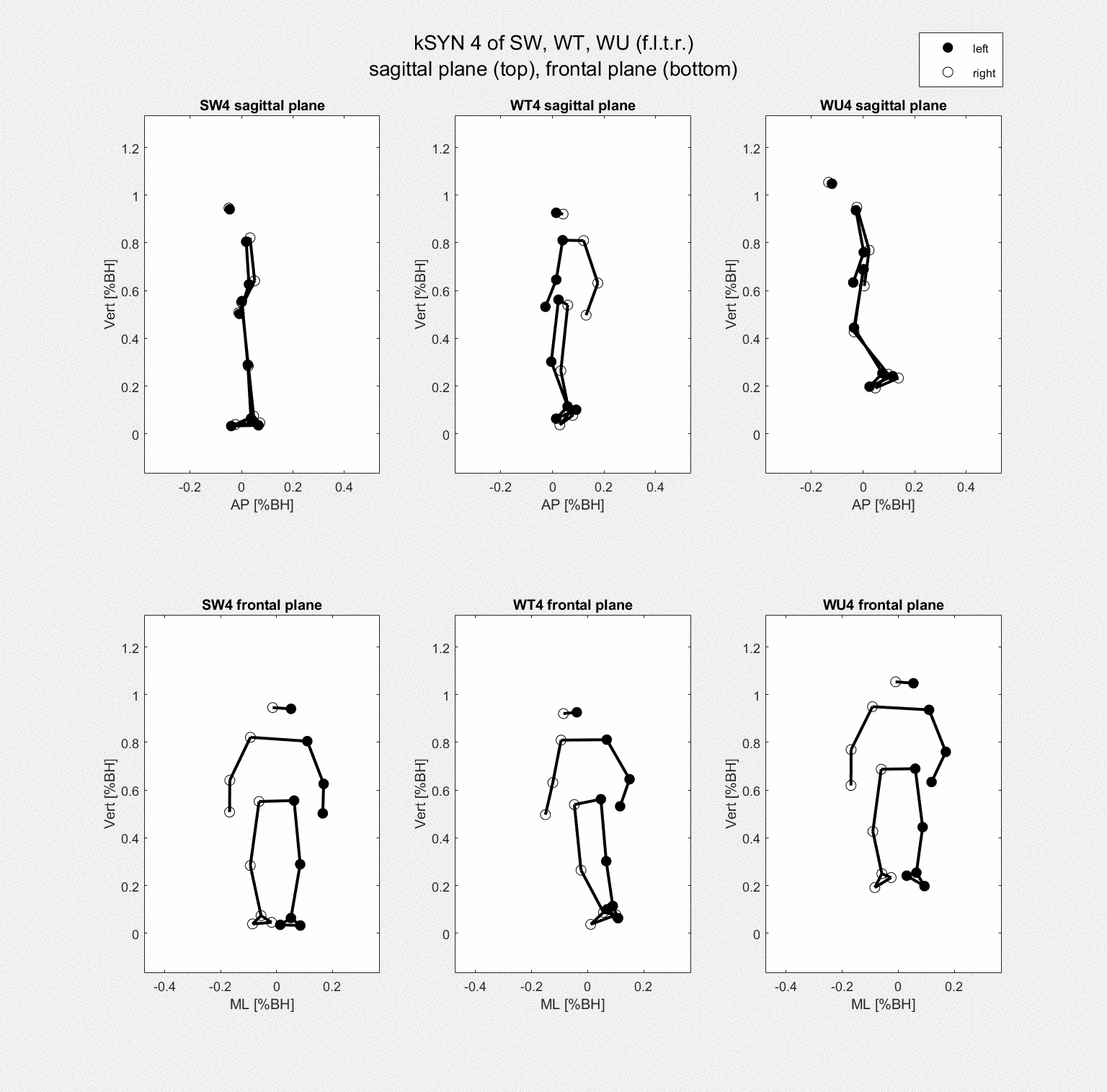

Supplement: Supplementary file 1 [file Data_Sheet_1.zip › kSYN4.gif]

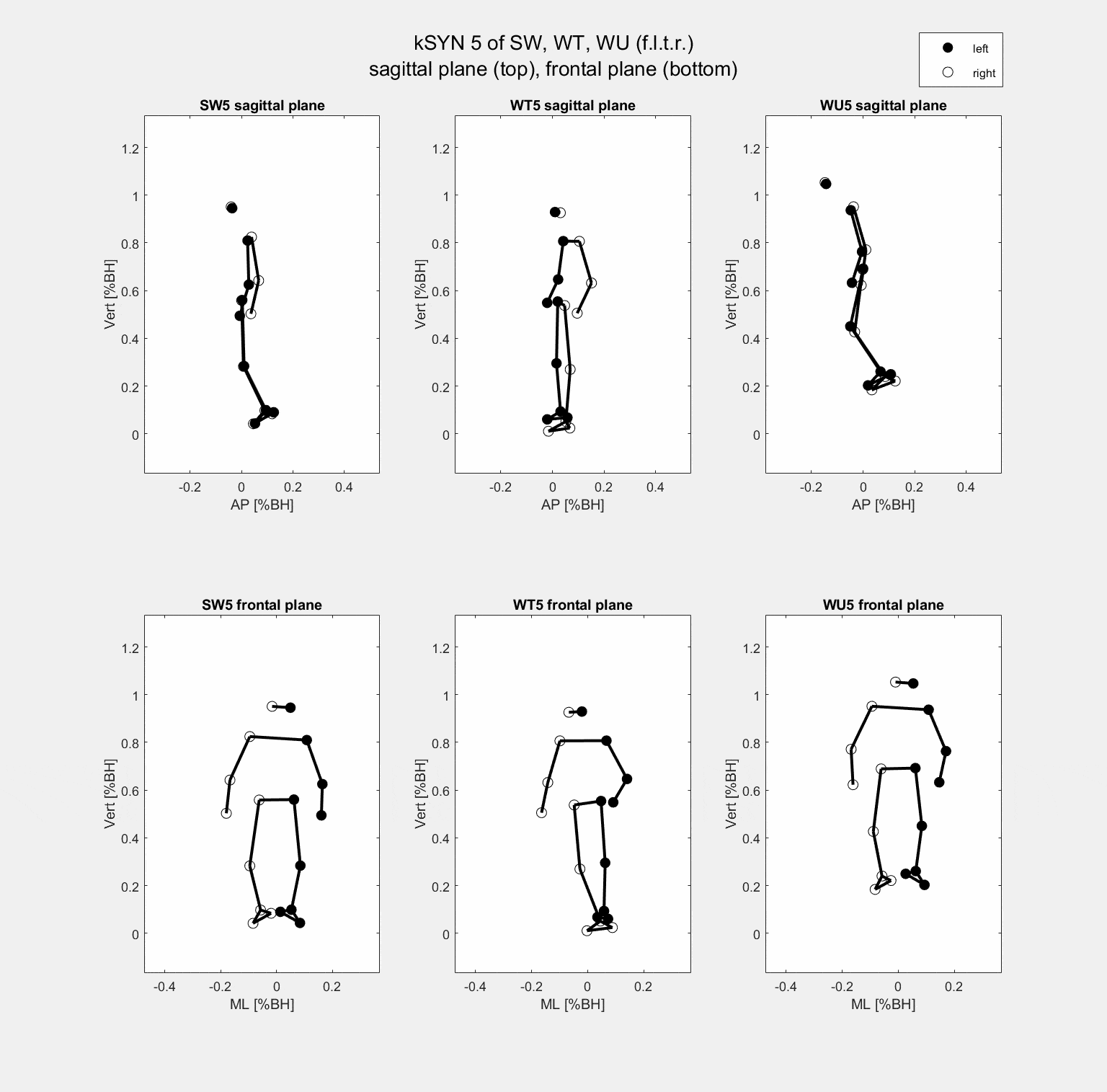

Supplement: Supplementary file 1 [file Data_Sheet_1.zip › kSYN5.gif]
